# Global PAC Bounds for Learning Discrete Time Markov Chains

**DOI:** 10.1007/978-3-030-53291-8_17

**Published:** 2020-06-16

**Authors:** Hugo Bazille, Blaise Genest, Cyrille Jegourel, Jun Sun

**Affiliations:** 8 Microsoft Research Lab, Redmond, WA USA; 9 University of Southern California, Los Angeles, CA USA; 10 Univ Rennes, CNRS & Rennes 1, Rennes, France; 11 Singapore University of Technology and Design, Singapore, Singapore; 12 Singapore Management University, Singapore, Singapore

## Abstract

Learning models from observations of a system is a powerful tool with many applications. In this paper, we consider learning Discrete Time Markov Chains (DTMC), with different methods such as *frequency estimation* or *Laplace smoothing*. While models learnt with such methods converge asymptotically towards the exact system, a more practical question in the realm of trusted machine learning is how accurate a model learnt with a limited time budget is. Existing approaches provide bounds on how close the model is to the original system, in terms of bounds on *local* (transition) probabilities, which has unclear implication on the *global* behavior.

In this work, we provide *global bounds on the error* made by such a learning process, in terms of global behaviors formalized using *temporal logic*. More precisely, we propose a learning process ensuring a bound on the error in the probabilities of these properties. While such learning process cannot exist for the full LTL logic, we provide one ensuring a bound that is uniform over all the formulas of CTL. Further, given one time-to-failure property, we provide an improved learning algorithm. Interestingly, frequency estimation is sufficient for the latter, while Laplace smoothing is needed to ensure non-trivial uniform bounds for the full CTL logic.

## Introduction

Discrete-Time Markov Chains (DTMC) are commonly used in model checking to model the behavior of stochastic systems [3, 4, 7, 26]. A DTMC is described by a set of states and transition probabilities between these states. The main issue with modeling stochastic systems using DTMCs is to obtain the transition probabilities. One appealing approach to overcome this issue is to observe the system and to *learn automatically* these transition probabilities [8, 30], e.g., using frequency estimation or Laplace (or additive) smoothing [12]. Frequency estimation works by observing a long run of the system and estimating each individual transition by its empirical frequency. However, in this case, the unseen transitions are estimated as zeros. Once the probability of a transition is set to zero, the probability to reach a state could be tremendously changed, e.g., from 1 to 0 if the probability of this transition in the system is small but non-zero. To overcome this problem, when the set of transitions with non-zero probability is known (but not their probabilities), Laplace smoothing assigns a positive probability to the unseen transitions, i.e., by adding a small quantity both to the numerator and the denominator of the estimate used in frequency estimation. Other smoothing methods exist, such as Good-Turing [15] and Kneser-Sey estimations [7], notably used in natural language processing. Notwithstanding smoothing generates estimation biases, all these methods converge asymptotically to the exact transition probabilities.

In practice, however, there is often limited budget in observing and learning from the system, and the validity of the learned model is in question. In trusted machine learning, it is thus crucial to measure how the learned model differs from the original system and to provide practical guidelines (e.g., on the number of observations) to guarantee some control of their divergence.

Comparing two Markov processes is a common problem that relies on a notion of divergence. Most existing approaches focus on deviations between the probabilities of local transitions (e.g., [5, 10, 27]). However, a single deviation in a transition probability between the original system and the learned model may lead to large differences in their global behaviors, even when no transitions are overlooked, as shown in our example 1. For instance, the probability of reaching certain state may be magnified by paths which go through the same deviated transition many times. It is thus important to use a measure that quantifies the differences over global behaviors, rather than simply checking whether the differences between the individual transition probabilities are low enough.

Technically, the knowledge of a lower bound on the transition probabilities is often assumed [1, 14]. While it is a soft assumption in many cases, such as when all transition probabilities are large enough, it is less clear how to obtain such a lower bound in other cases, such as when a very unlikely transition exists (e.g., a very small error probability). We show how to handle this in several cases: learning a Markov chain accurate w.r.t. this error rate, or learning a Markov chain accurate over all its global behaviors, which is possible if we know the underlying structure of the system (e.g., because we designed it, although we do not know the precise transition probabilities which are governed by uncertain forces). For the latter, we define a new concept, namely *conditioning* of a DTMC.

In this work, we model global behaviors using temporal logics. We consider Linear Temporal Logic (LTL) [24] and Computational Tree Logic (CTL) [11]. Agreeing on all formulas of LTL means that the first order behaviors of the system and the model are the same, while agreeing on CTL means that the system and the model are bisimilar [2]. Our goal is to provide stopping rules in the learning process of DTMCs that provides Probably Approximately Correct (PAC) bounds on the error in probabilities of every property in the logic between the model and the system. In Sect. 2, we recall useful notions on DTMCs and PAC-learning. We point out related works in Sect. 3. Our main contributions are as follows:

- In Sect. 4, we show that it is impossible to learn a DTMC accurate for all LTL formulas, by adapting a result from [13].
- We provide in Sect. 6 a learning process bounding the difference in probability *uniformly over all CTL properties*. To do so, we use Laplace smoothing, and we provide rationale on choosing the smoothing parameter.
- For the particular case of a time-to-failure property, notably used to compute the mean time between failures of critical systems (see e.g., [25]), we provide tighter bounds in Sect. 5, based on frequency estimation.

In Sect. 4, we formally state the problem and the specification that the learning process must fulfill. We also show our first contribution: the impossibility of learning a DTMC, accurate for all LTL formulas. Nevertheless, we prove in Sect. 5 our second contribution: the existence of a global bound for the time-to-failure properties, notably used to compute the mean time between failures of critical systems (see e.g., [25]) and provide an improved learning process, based on frequency estimation. In Sect. 6, we present our main contribution: a global bound guaranteeing that the original system and a model learned by Laplace smoothing have similar behaviors for all the formulas in CTL. We show that the error bound that we provide on the probabilities of properties is close to optimal. We evaluate our approach in Sect. 7 and conclude in Sect. 8.

## Background

In this section, we introduce the notions and notations used throughout the paper. A stochastic system $\mathcal {S}$ is interpreted as a set of interacting components in which the state is determined randomly with respect to a global probability measure described below.

### Definition 1 (Discrete-Time Markov Chains)

A Discrete-Time Markov Chain is a triple $\mathcal {M}=\left( S,\mu ,A\right)$ where:

- *S* is a finite set of states;
- $\mu : S\rightarrow \left[ 0,1\right]$ is an initial probability distribution over *S*;
- $A: S\times S\rightarrow \left[ 0,1\right]$ is a transition probability matrix, such that for every $s\in S$, $\sum _{s'\in S}A(s,s')=1$.

**Fig. 1.**
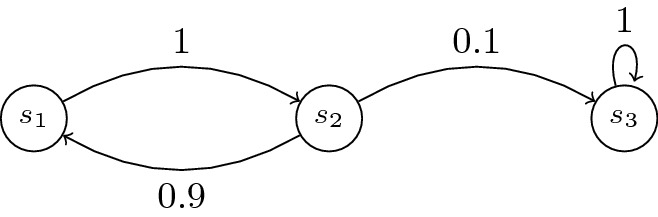
An example of DTMC $\mathcal{M}_1$

**Fig. 2.**
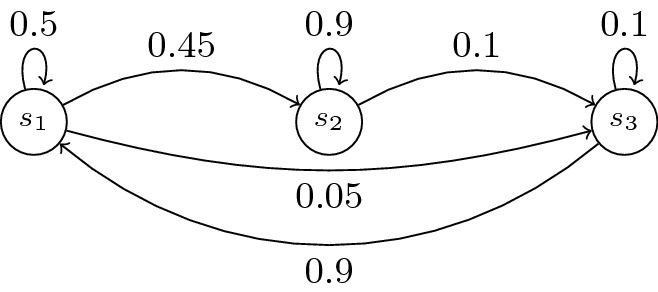
DTMC $$\mathcal{M}_2$$

We denote by *m* the cardinal of *S* and $A=(a_{ij})_{1\le i,j\le m}=(A(i,j))_{1\le i,j\le m}$ the probability matrix. Figures 1 and 2 show the graph of two DTMCs over 3 states $\{s_1,s_2,s_3\}$ (with $\mu (s_1)=1$). A run is an infinite sequence $\omega =s_0s_1\cdots$ and a path is a finite sequence $\omega =s_0\cdots s_l$ such that $\mu (s_0)>0$ and $A(s_i,s_{i+1})>0$ for all *i*, $0\le i\le l$. The length $|\omega |$ of a path $\omega$ is its number of transitions.

The cylinder set of $\omega$, denoted $C(\omega )$, consists of all the runs starting by a path $\omega$. Markov chain $\mathcal {M}$ underlies a probability space $(\varOmega ,\mathcal {F},\mathbb {P})$, where $\varOmega$ is the set of all runs from $\mathcal {M}$; $\mathcal {F}$ is the sigma-algebra generated by all the cylinders $C(\omega )$ and $\mathbb {P}$ is the unique probability measure [32] such that $\mathbb {P}(C(s_0\cdots s_l))=\mu (s_0)\prod _{i=1}^l A(s_{i-1},s_{i})$. For simplicity, we assume a unique initial state $s_0$ and denote $\mathbb {P}(\omega )=\mathbb {P}\left( C(\omega )\right)$. Finally, we sometimes use the notation $\mathbb {P}^A_i$ to emphasize that the probability distribution is parameterized by the probability matrix *A*, and the starting state is *i*.

### PAC-Learning for Properties

To analyze the behavior of a system, properties are specified in temporal logic (e.g., LTL or CTL, respectively introduced in [24] and [11]). Given a logic $\mathcal {L}$ and $\varphi$ a property of $\mathcal {L}$, decidable in finite time, we denote $\omega \models \varphi$ if a path $\omega$ satisfies $\varphi$. Let $z:\varOmega \times \mathcal {L}\rightarrow \{0,1\}$ be the function that assigns 1 to a path $\omega$ if $\omega \models \varphi$ and 0 otherwise. In what follows, we assume that we have a procedure that draws path $\omega$ with respect to $\mathbb {P}^A$ and outputs $z(\omega ,\varphi )$. Further, we denote $\gamma (A,\varphi )$ the probability that a path drawn with respect to $\mathbb {P}^A$ satisfies $\varphi$. We omit the property or the matrix in the notation when it is clear from the context. Finally, note that the behavior of $z(.,\varphi )$ can be modeled as a Bernoulli random variable $Z_{\varphi }$ parameterized by the mean value $\gamma (A,\varphi )$.

Probably Approximately Correct (PAC) learning [28] is a framework for mathematical analysis of machine learning. Given $\varepsilon >0$ and $0<\delta <1$, we say that a property $\varphi$ of $\mathcal {L}$ is PAC-learnable if there is an algorithm $\mathcal {A}$ such that, given a sample of *n* paths drawn according to the procedure, with probability of at least $1-\delta$, $\mathcal {A}$ outputs in polynomial time (in $1/\varepsilon$ and $1/\delta$) an approximation of the average value for $Z_{\varphi }$ close to its exact value, up to an error less than or equal to $\varepsilon$. Formally, $\varphi$ is PAC-learnable if and only if $\mathcal {A}$ outputs an approximation $\hat{\gamma }$ such that:

1 $$\begin{aligned} \mathbb {P}\left( |\gamma -\hat{\gamma }|>\varepsilon \right) \le \delta \end{aligned}$$

Moreover, if the above statement for algorithm $\mathcal {A}$ is true for every property in $\mathcal {L}$, we say that $\mathcal {A}$ is a PAC-learning algorithm for $\mathcal {L}$.

### Monte-Carlo Estimation and Algorithm of Chen

Given a sample *W* of *n* paths drawn according to $\mathbb {P}^A$ until $\varphi$ is satisfied or violated (for $\varphi$ such that with probability 1, $\varphi$ is eventually satisfied or violated), the crude Monte-Carlo estimator, denoted $\hat{\gamma }_W(A,\varphi )$, of the mean value for the random variable $Z_{\varphi }$ is given by the empirical frequency: $\hat{\gamma }_W(A,\varphi )=\frac{1}{n}\sum _{i=1}^{n}z(\omega _{i})\approx \gamma (A,\varphi )$.

The Okamoto inequality [23] (also called the Chernoff bound in the literature) is often used to guarantee that the deviation between a Monte-Carlo estimator $\hat{\gamma }_W$ and the exact value $\gamma$ by more than $\varepsilon >0$ is bounded by a predefined confidence parameter $\delta$. However, several sequential algorithms have been recently proposed to guarantee the same confidence and accuracy with fewer samples^1^. In what follows, we use the Massart bound [22], implemented in the algorithm of Chen [6].

#### Theorem 1 (Chen bound)

Let $\varepsilon >0$, $\delta$ such that $0<\delta <1$ and $\hat{\gamma }_W$ be the crude Monte-Carlo estimator, based on *n* samples, of probability $\gamma$.

If $$n\ge \frac{2}{\varepsilon ^2}\log \left( \frac{2}{\delta }\right) \left[ \frac{1}{4}-(|\frac{1}{2}-\hat{\gamma }_W|-\frac{2}{3}\varepsilon )^2\right] $$,

$$\begin{aligned} \mathbb {P}(|\gamma -\hat{\gamma }_W| >\varepsilon )\le \delta . \end{aligned}$$

To ease the readability, we write $n_{\text {succ}}=\sum _{i=1}^n z(\omega _i)$ and $H(n,n_{\text {succ}},\epsilon ,\delta )=\frac{2}{\varepsilon ^2}\log \left( \frac{2}{\delta }\right) \left[ \frac{1}{4}-(|\frac{1}{2}-\hat{\gamma }_W|-\frac{2}{3}\varepsilon )^2\right]$. When it is clear from the context, we only write *H*(*n*). Then, the algorithm $\mathcal {A}$ that stops sampling as soon as $n\ge H(n)$ and outputs a crude Monte-Carlo estimator for $\gamma (A,\varphi )$ is a PAC-learning algorithm for $\varphi$. The condition over *n* is called the stopping criteria of the algorithm. As far as we know, this algorithm requires fewer samples than the other sequential algorithms (see e.g., [18]). Note that the estimation of a probability close to 1/2 likely requires more samples since *H*(*n*) is maximized in $\hat{\gamma }_W=1/2$.

## Related Work

Our work shares similar statistical results (see Sect. 2.3) with Statistical Model Checking (SMC) [32]. However, the context and the outputs are different. SMC is a simulation-based approach that aims to estimate one probability for a given property [9, 29], within acceptable margins of error and confidence [17, 18, 33]. A challenge in SMC is posed by unbounded properties (e.g., fairness) since the sampled executions are finite. Some algorithms have been proposed to handle unbounded properties but they require the knowledge of the minimal probability transition of the system [1, 14], which we avoid. While this restriction is light in many contexts, such as when every state and transition appears with a sufficiently high probability, contexts where probabilities are unknown and some are very small seems much harder to handle. In the following, we propose 2 solutions not requiring this assumption. The first one is the closest to SMC: we learn a Markov chain accurate for a given time-to-error property, and it does not require knowledge on the Markov chain. The second one is much more ambitious than SMC as it learns a Markov chain accurate for *all* its global behaviors, formalized as all properties of a temporal logic; it needs the assumption that the set of transitions is known, but not their probabilities nor a lower bound on them. This assumption may seem heavy, but it is reasonable for designers of systems, for which (a lower bound on) transition probabilities are not known (e.g. some error rate of components, etc).

For comparison with SMC, our final output is the (approximated) transition matrix of a DTMC rather than one (approximated) probability of a given property. This learned DTMC can be used for different purposes, e.g. as a component in a bigger model or as a simulation tool. In terms of performances, we will show that we can learn a DTMC w.r.t. a given property with the same number of samples as we need to estimate this property using SMC (see Sect. 5). That is, there is no penalty to estimate a DTMC rather than estimate one probability, and we can scale as well as SMC. In terms of expressivity, we can handle unbounded properties (e.g. fairness properties). Even better, we can learn a DTMC accurate uniformly over a possibly infinite set of properties, e.g. all formulas of CTL. This is something SMC is not designed to achieve.

Other related work can be cited: In [13], the authors investigate several distances for the estimation of the difference between DTMCs. But they do not propose algorithms for learning. In [16], the authors propose to analyze the learned model a posteriori to test whether it has some good properties. If not, then they tweak the model in order to enforce these properties. Also, several PAC-learning algorithms have been proposed for the estimation of stochastic systems [5, 10] but these works focus on local transitions instead of global properties.

## Problem Statement

In this work, we are interested to learn a DTMC model from a stochastic system ${\mathcal S}$ such that the behaviors of the system and the model are similar. We assume that the original system is a DTMC parameterized by a matrix *A* of transition probabilities. The transition probabilities are unknown, but the set of states of the DTMC is assumed to be known.

Our goal is to provide a learning algorithm $\mathcal {A}$ that guarantees an accurate estimation of ${\mathcal S}$ with respect to certain global properties. For that, a sampling process is defined as follows. A path (i.e., a sequence of states from $s_0$) of ${\mathcal S}$ is observed, and at steps specified by the sampling process, a reset action is performed, setting ${\mathcal S}$ back to its initial state $s_0$. Then another path is generated. This process generates a set *W* of paths, called traces, used to learn a matrix $\hat{A}_W$. Formally, we want to provide a learning algorithm that guarantees the following specification:

2 $$\begin{aligned} \mathbb {P}(\mathcal {D}(A,\hat{A}_W)>\varepsilon )\le \delta \end{aligned}$$

where $\varepsilon >0$ and $\delta >0$ are respectively *accuracy* and *confidence* parameters and $\mathcal {D}(A,\hat{A}_W)$ is a measure of the divergence between *A* and $\hat{A}_W$.

There exist several ways to specify the divergence between two transition matrices, e.g., the Kullback-Leibler divergence [19] or a distance based on a matrix norm. However, the existing notions remain heuristic because they are based on the difference between the individual probabilistic transitions of the matrix. We argue that what matters in practice is often to quantify the similarity between the global behaviors of the systems and the learned model.

In order to specify the behaviors of interest, we use a property $\varphi$ or a set of properties $\varPsi$ on the set of states visited. We are interested in the difference between the probabilities of $\varphi$ (i.e., the measure of the set of runs satisfying $\varphi$) with respect to *A* and $\hat{A}_W$. We want to ensure that this difference is less than some predefined $\varepsilon$ with (high) probability $1-\delta$. Hence, we define:

3 $$\begin{aligned} \mathcal {D_{\varphi }}(A,\hat{A}_W)&= |\gamma (A,\varphi )-\gamma (\hat{A}_W,\varphi )| \end{aligned}$$

4 $$\begin{aligned} \mathcal {D}_{\varPsi }(A,\hat{A}_W)&= \max _{\varphi \in \varPsi }(\mathcal {D_{\varphi }}(A,\hat{A}_W)) \end{aligned}$$

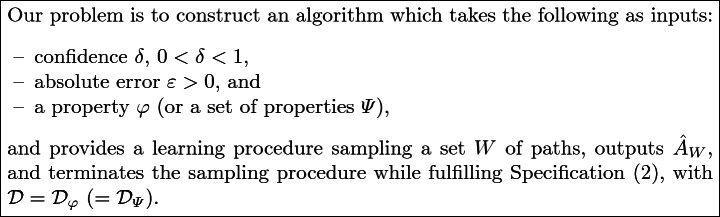


In what follows, we assume that the confidence level $\delta$ and absolute error $\varepsilon$ are fixed. We first start with a negative result: if $\varPsi$ is the set of LTL formulas [2], such a learning process is impossible.

### Theorem 2

Given $\varepsilon >0$, $0<\delta <1$, and a finite set *W* of paths randomly drawn with respect to a DTMC *A*, there is no learning strategy such that, for every LTL formula $\varphi$,

5 $$\begin{aligned} \mathbb {P}(|\gamma (A,\varphi ) - \gamma (\hat{A}_W,\varphi )| >\varepsilon )\le \delta \end{aligned}$$

Note that contrary to Theorem 1, the deviation in Theorem 2 is a difference between two exact probabilities (of the original system and of a learned model). The theorem holds as long as $\hat{A}_W$ and *A* are not strictly equal, no matter how $\hat{A}_W$ is learned. To prove this theorem, we show that, for any number of observations, we can always define a sequence of LTL properties that violates the specification above. It only exploits a single deviation in one transition. The proof, inspired by a result from [13], is given in the extended version.

### Example 1

We show in this example that in general, one needs to have some knowledge on the system in order to perform PAC learning - either a positive lower bound $\ell >0$ on the lowest probability transition, as in [1, 14], or the support of transitions (but no knowledge on their probabilities), as we use in Sect. 6. Further, we show that the latter assumption does not imply the former, as even if no transitions are overlooked, the error in some reachability property can be arbitrarily close to 0.5 even with arbitrarily small error on the transition probabilities.

**Fig. 3.**
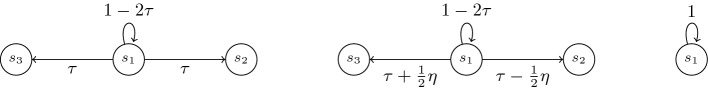
Three DTMCs $A,\hat{A},\hat{B}$ (from left to right), with $0< \eta< 2 \tau < 1$

Let us consider DTMCs $A,\hat{A},\hat{B}$ in Fig. 3, and formula $\mathbf {F} \,s_2$ stating that $s_2$ is eventually reached. The probabilities to satisfy this formula in $A,\hat{A},\hat{B}$ are respectively $\mathbb {P}^{A}(\mathbf {F} \,s_2)= \frac{1}{2}$, $\mathbb {P}^{\hat{A}}(\mathbf {F} \,s_2)= \frac{2\tau -\eta }{4\tau }=\frac{1}{2} - \frac{\eta }{4\tau }$ and $\mathbb {P}^{\hat{B}}(\mathbf {F} \,s_2)=0$.

Assume that *A* is the real system and that $\hat{A}$ and $\hat{B}$ are DTMCs we learned from *A*. Obviously, one wants to avoid learning $\hat{B}$ from *A*, as the probability of $\mathbf {F} \,s_2$ is very different in $\hat{B}$ and in $\hat{A}$ (0 instead of 0.5). If one knows that $\tau > \ell$ for some lower bound $\ell >0$, then one can generate enough samples from $s_1$ to evaluate $\tau$ with an arbitrarily small error $\frac{\eta }{2}<< \ell$ on probability transitions with an arbitrarily high confidence, and in particular learn a DTMC similar to $\hat{A}$.

On the other hand, if one knows there are transitions from $s_1$ to $s_2$ and to $s_3$, then immediately, one does not learn DTMC $\hat{B}$, but a DTMC similar to DTMC $\hat{A}$ (using e.g. Laplace smoothing [12]). While this part is straightforward with this assumption, evaluating $\tau$ is much harder when one does not know a priori a lower bound $\ell >0$ such that $\tau > \ell$. That is very important: while one can make sure that the error $\frac{\eta }{2}$ on probability transitions is arbitrarily small, if $\tau$ is unknown, then it could be the case that $\tau$ is as small as $\frac{\eta }{2(1-\varepsilon )}>\frac{\eta }{2}$, for a small $\varepsilon >0$. This gives us $\mathbb {P}^{\hat{A}}(\mathbf {F} \,s_2)= \frac{1}{2} - \frac{1-\varepsilon }{2}=\frac{\varepsilon }{2}$, which is arbitrarily small, whereas $\mathbb {P}^{A}(\mathbf {F} \,s_2)=0.5$, leading to a huge error in the probability to reach $s_2$. We work around that problem in Sect. 6 by defining and computing the *conditioning* of DTMC $\hat{A}$. In some particular cases, as the one discussed in the next section, one can avoid that altogether (actually, the conditioning in these cases is perfect (=1), and it needs not be computed explicitly).

## Learning for a Time-to-failure Property

In this section, we focus on property $\varphi$ of reaching a failure state $s_F$ from an initial state $s_0$ without re-passing by the initial state, which is often used for assessing the failure rate of a system and the mean time between failures (see e.g., [25]). We assume that with probability 1, the runs eventually re-pass by $s_0$ or reach $s_F$. Also, without loss of generality, we assume that there is a unique failure state $s_F$ in *A*. We denote $\gamma (A,\varphi )$ the probability, given DTMC *A*, of satisfying property $\varphi$, i.e., the probability of a failure between two visits of $s_0$.

Assume that the stochastic system $\mathcal {S}$ is observed from state $s_0$. Between two visits of $s_0$, property $\varphi$ can be monitored. If $s_F$ is observed between two instances of $s_0$, we say that the path $\omega =s_0 \cdot \rho \cdot s_F$ satisfies $\varphi$, with $s_0,s_F \notin \rho$. Otherwise, if $s_0$ is visited again from $s_0$, then we say that the path $\omega =s_0 \cdot \rho \cdot s_0$ violates $\varphi$, with $s_0,s_F \notin \rho$. We call *traces* paths of the form $\omega = s_0 \cdot \rho \cdot (s_0 \vee s_F)$ with $s_0,s_F \notin \rho$. In the following, we show that it is sufficient to use a *frequency estimator* to learn a DTMC which provides a good approximation for such a property.

### Frequency Estimation of a DTMC

Given a set *W* of *n* traces, we denote $n^W_{ij}$ the number of times a transition from state *i* to state *j* has occurred and $n^W_i$ the number of times a transition has been taken from state *i*.

The *frequency estimator* of *A* is the DTMC $\hat{A}_W=(\hat{a}_{ij})_{1\le i,j\le m}$ given by $\hat{a}_{ij}= \frac{n^W_{ij}}{n^W_i}$ for all *i*, *j*, with $\sum _{i=1}^m n^W_i=\sum _{i=1}^m\sum _{j=1}^m n^W_{ij}=|W|$. In other words, to learn $\hat{A}_W$, it suffices to count the number of times a transition from *i* to *j* occurred, and divide by the number of times state *i* has been observed. The matrix $\hat{A}_W$ is trivially a DTMC, except for states *i* which have not been visited. In this case, one can set $\hat{a}_{ij}=\frac{1}{m}$ for all states *j* and obtain a DTMC. This has no impact on the behavior of $\hat{A}_W$ as *i* is not reachable from $s_0$ in $\hat{A}_W$.

Let $\hat{A}_W$ be the matrix learned using the frequency estimator from the set *W* of traces, and let *A* be the real probabilistic matrix of the original system $\mathcal {S}$. We show that, in the case of time-to-failure properties, $\gamma (\hat{A}_W,\varphi )$ is equal to the crude Monte Carlo estimator $\hat{\gamma }_W(A,\varphi )$ induced by *W*.

### PAC Bounds for a Time-to-failure Property

We start by stating the main result of this section, bounding the error between $\gamma (A,\varphi )$ and $\gamma (\hat{A}_W,\varphi )$:

#### Theorem 3

Given a set *W* of *n* traces such that $n=\lceil H(n)\rceil$, we have:

6 $$\begin{aligned} \mathbb {P}\left( |\gamma (A,\varphi ) - \gamma (\hat{A}_W,\varphi )| >\varepsilon \right) \le \delta \end{aligned}$$

where $\hat{A}_W$ is the frequency estimator of *A*.

To prove Theorem (3), we first invoke Theorem 1 to establish:

7 $$\begin{aligned} \mathbb {P}\left( |\gamma (A,\varphi ) - {\hat{\gamma }_W}(A,\varphi )| > \varepsilon \right) \le \delta \end{aligned}$$

It remains to show that $\hat{\gamma }_W(A,\varphi ) = \gamma (\hat{A}_W,\varphi )$:

#### Proposition 1

Given a set *W* of traces, $\gamma (\hat{A}_W,\varphi ) = \hat{\gamma }_W(A,\varphi )$.

It might be appealing to think that this result can be proved by induction on the size of the traces, mimicking the proof of computation of reachability probabilities by linear programming [2]. This is actually not the case. The remaining of this section is devoted to proving Proposition (1).

We first define $q_{W}(u)$ the number of occurrences of sequence *u* in the traces of *W*. Note that *u* can be a state, an individual transition or even a path. We also use the following definitions in the proof.

#### Definition 2 (Equivalence)

Two sets of traces *W* and $W'$ are equivalent if for all $s,t\in S$, $\frac{q_W(s\cdot t)}{q_W(s)}=\frac{q_{W'}(s\cdot t)}{q_{W'}(s)}$.

We define a set of traces $W'$ equivalent with *W*, implying that $\hat{A}_W =\hat{A}_{W'}$. This set $W'$ of traces satisfies the following:

#### Lemma 1

For any set of traces *W*, there exists a set of traces $W'$ such that:

(i) *W* and $W'$ are equivalent,
(ii) for all $r,s,t\in S$, $\displaystyle q_{W'}(r \cdot s \cdot t) = \frac{q_{W'}(r \cdot s)\times q_{W'}(s \cdot t)}{q_{W'}(s)}$.

The proof of Lemma 1 is provided in the extended version. In Lemma 1, (i) ensures that $\hat{A}_{W'} =\hat{A}_{W}$ and (ii) ensures the equality between the proportion of runs of $W'$ passing by *s* and satisfying $\gamma$, denoted $\hat{\gamma }^s_{W'}$, and the probability of reaching $s_F$ before $s_0$ starting from *s* with respect to $\hat{A}_{W'}$. Formally,

#### Lemma 2

For all $$s \in S$$, $$\mathbb {P}^{\hat{A}_{W'}}_s(\text {reach }s_f\text { before }s_0) = \hat{\gamma }^s_{W'}$$.

#### Proof

Let $S_0$ be the set of states *s* with no path in $\hat{A}_{W'}$ from *s* to $s_f$ without passing through $s_0$. For all $s \in S_0$, let $p_s = 0$. Also, let $p_{s_f} = 1$. Let $S_1= S \setminus (S_0 \cup \{s_f\})$. Consider the system of Eq. (8) with variables $(p_s)_{s \in S_1} \in [0,1]^{|S_1|}$:

8 $$\begin{aligned} \forall \,s \in S_1,\quad p_s=\sum _{t=1}^m \hat{A}_{W'}(s,t)p_t \end{aligned}$$

The system of Eq. (8) admits a unique solution according to [2] (Theorem 10.19. page 766). Then, $(\mathbb {P}^{\hat{A}_{W'}}_s(\text {reach }s_f\text { before }s_0))_{s \in S_1}$ is trivially a solution of (8). But, since $W'$ satisfies the conditions of Lemma 1, we also have that $(\hat{\gamma }^s_{W'})_{s \in S_1}$ is a solution of (8), and thus we have the desired equality.$\square$

Notice that Lemma 2 does not hold in general with the set *W*. We have:

$$\begin{aligned} \hat{\gamma }_W(A,\varphi )&= \hat{\gamma }^{s_0}_{W}\quad \text {(by definition)}\\&=\hat{\gamma }^{s_0}_{W'}\quad \text {(by Lemma~1)}\\&=\mathbb {P}^{\hat{A}_{W'}}_{s_0}(\text {reach }s_f\text { before }s_0)\quad \text {(by Lemma~2)}\\&=\mathbb {P}^{\hat{A}_{W}}_{s_0}(\text {reach }s_f\text { before }s_0)\quad \text {(by Lemma~1)}\\&=\gamma (\hat{A}_{W},\varphi )\quad \text {(by definition)}. \end{aligned}$$

That concludes the proof of Proposition 1. It shows that learning can be as efficient as statistical model-checking on comparable properties.

## Learning for the Full CTL Logic

In this section, we learn a DTMC $\hat{A}_W$ such that $\hat{A}_W$ and *A* have similar behaviors over all CTL formulas. This provides a much stronger result than on time-to-failure property, e.g., properties can involve liveness and fairness, and more importantly they are not known before the learning. Notice that PCTL [2] cannot be used, since an infinitesimal error on one ${>}\,0$ probability can change the probability of a PCTL formula from 0 to 1. (State)-CTL is defined as follows:

### Definition 3

Let *Prop* be the set of state names. (State)-CTL is defined by the following grammar $\varphi \,{::=}\,\bot \,\mid \top \,\mid p\,\mid \lnot \varphi \,\mid \varphi \wedge \varphi \,\mid \varphi \vee \varphi \,\mid \varphi \wedge \varphi \,\mid \mathbf {AX} \varphi \,\mid \mathbf {EX} \varphi \,\mid \mathbf {AF} \varphi \,\mid \mathbf {EF} \varphi \,\mid \mathbf {AF} \varphi \,\mid \mathbf {EG} \varphi \,\mid \mathbf {AG} \varphi \,\mid \mathbf {E}(\varphi \mathbf {U}\varphi )\,\mid \mathbf {A}(\varphi \mathbf {U}\varphi )$, with $p\in Prop$. $\mathbf {E}$(xists) and $\mathbf {A}$(ll) are quantifiers on paths, ne$\mathbf {X}$t, $\mathbf {G}$lobally, $\mathbf {F}$inally and $\mathbf {U}$ntil are path-specific quantifiers. Notice that some operators are redundant. A minimal set of operators is $\{\top , \vee , \lnot , \mathbf {EG,EU,EX}\}$.

As we want to compute the probability of *paths* satisfying a CTL formula, we consider the set $\varPsi$ of *path-CTL* properties, that is formulas $\varphi$ of the form $\varphi =\mathbf {X} \varphi _1$, $\varphi =\varphi _1 \mathbf {U} \varphi _2$, $\varphi =\mathbf {F} \varphi _1$ or $\varphi =\mathbf {G} \varphi _1$, with $\varphi _1,\varphi _2$ (state)-CTL formulas. For instance, the property considered in the previous section is $(\lnot s_0) \mathbf {U} s_F$.

In this section, for the sake of simplicity, the finite set *W* of traces is obtained by observing paths till a state is seen twice on the path. Then, the reset action is used and another trace is obtained from another path. That is, a trace $\omega$ from *W* is of the form $\omega =\rho \cdot s \cdot \rho ' \cdot s$, with $\rho \cdot s\cdot \rho '$ a loop-free path.

As explained in example 1, some additional knowledge on the system is necessary. In this section, we assume that the support of transition probabilities is known, i.e., for any state *i*, we know the set of states *j* such that $a_{ij} \ne 0$. This assumption is needed both for Theorem 5 and to apply Laplace smoothing.

### Learning DTMCs with Laplace Smoothing

Let $\alpha >0$. For any state *s*, let $k_s$ be the number of successors of *s*, that we know by hypothesis, and $T=\sum _{s\in S}k_s$ be the number of non-zero transitions. Let *W* be a set of traces, $n^W_{ij}$ the number of transitions from state *i* to state *j*, and $n^W_i = \sum _j n^W_{ij}$. The *estimator for W* *with Laplace smoothing *$\alpha$ is the DTMC $\hat{A}^\alpha _W=(\hat{a}_{ij})_{1\le i,j\le m}$ given for all *i*, *j* by:

$$\hat{a}_{ij}= \frac{n^W_{ij}+\alpha }{n^W_i+k_i \alpha } \text { if } a_{ij} \ne 0 \quad \text { and } \quad \hat{a}_{ij}=0 \text { otherwise}$$

In comparison with the frequency estimator, the Laplace smoothing adds for each state *s* a term $\alpha$ to the numerator and $k_s$ times $\alpha$ to the denominator. This preserves the fact that $\hat{A}^\alpha _W$ is a Markov chain, and it ensures that $\hat{a}_{ij} \ne 0$ iff $a_{ij} \ne 0$. In particular, compared with the frequency estimator, it avoids creating zeros in the probability tables.

### Conditioning and Probability Bounds

Using Laplace smoothing slightly changes the probability of each transition by an additive offset $\eta$. We now explain how this small error $\eta$ impacts the error on the probability of a CTL property.

Let *A* be a DTMC, and $A_{\eta }$ be a DTMC such that $A_{\eta }(i,j) \ne 0$ iff $A(i,j) \ne 0$ for all states *i*, *j*, and such that $\sum _j |A_{\eta }(i,j) - A(i,j)| \le \eta$ for all states *i*. For all states $s \in S$, let *R*(*s*) be the set of states *i* such that there exists a path from *i* to *s*. Let $R_*(s) = R(s) \setminus \{s\}$. Since both DTMCs have the same support, *R* (and also $R_*$) is equal for *A* and $A_{\eta }$. Given *m* the number of states, the conditioning of *A* for $s \in S$ and $\ell \le m$ is:

$$\begin{aligned} \text {Cond}_s^\ell (A)=\min _{i \in R_*(s)} \mathbb {P}^A_i(\mathbf {F}_{\le \ell } \lnot R_*(s)) \end{aligned}$$

i.e., the minimal probability from state $i \in R_*(s)$ to move away from $R_*(s)$ in at most $\ell$ steps. Let $\ell _s$ be the minimal value such that $\text {Cond}^{\ell _s}_s(A)>0$. This minimal $\ell _s$ exists as $\text {Cond}^m_s(A)>0$ since, for all $s\in S$ and $i \in R_*(s)$, there is at least one path reaching *s* from *i* (this path leaves $R_*(s)$), and taking a cycle-free path, we obtain a path of length at most *m*. Thus, the probability $\mathbb {P}^A_i(\mathbf {F}_{ \le m} \lnot R_*(s))$ is at least the positive probability of the cylinder defined by this finite path. Formally,

#### Theorem 4

Denoting $\varphi$ the property of reaching state *s* in DTMC *A*, we have:

$$\begin{aligned} |\gamma (A,\varphi )-\gamma (A_{\eta },\varphi )| < \frac{\ell _s \cdot \eta }{\text {Cond}_s^{\ell _s}(A)} \end{aligned}$$

#### Proof

Let $v_s$ be the stochastic vector with $v_s(s)=1$. We denote $v_0=v_{s_0}$. Let $s \in S$. We assume that $s_0 \in R_*(s)$ (else $\gamma (A,\varphi )=\gamma (A_\eta ,\varphi )$ and the result is trivial). Without loss of generality, we can also assume that $A(s,s)=A_{\eta }(s,s)=1$ (as we are interested in reaching *s* at any step). With this assumption:

$$|\gamma (A,\varphi )-\gamma (A_{\eta },\varphi )| = \lim _{t \rightarrow \infty } |v_0 \cdot (A^t-A_{\eta }^t) \cdot v_s|$$

We bound this error, through bounding by induction on *t*:

$$E(t)= \max _{i \in R_*(s)} |v_i \cdot (A^t-A_{\eta }^t) \cdot v_s|$$

We then have trivially:

$$|\gamma (A,\varphi )-\gamma (A_{\eta },\varphi )| \le \lim _{t \rightarrow \infty } E(t)$$

Note that for $i=s$, $\lim _{t \rightarrow \infty } v_i \cdot (A^t) \cdot v_s = 1 = \lim _{t \rightarrow \infty } v_i \cdot A_{\eta }^t \cdot v_s$, and thus their difference is null.

Let $t \in \mathbb {N}$. We let $j \in R_*(s)$ such that $E(t)= |v_j \cdot (A^t-A_{\eta }^t) \cdot v_s|$.

By the triangular inequality, introducing the term $v_j \cdot A^{\ell _s} A_{\eta }^{t-k} \cdot v_s - v_j \cdot A^{\ell _s} A_{\eta }^{t-k} \cdot v_s = 0$, we have:

$$\begin{aligned} E(t)&\le |v_j \cdot (A_{\eta }^{t}- A^{\ell _s} A_{\eta }^{t-\ell _s}) \cdot v_s| + |(v_j \cdot A^{\ell _s}) \cdot (A_{\eta }^{t-\ell _s} - A^{t-\ell _s}) \cdot v_s| \end{aligned}$$

We separate vector $(v_j \cdot A^{\ell _s})=w_1+w_2+w_3$ in three sub-stochastic vectors $w_1,w_2,w_3$: vector $w_1$ is over $\{s\}$, and thus we have $w_1\cdot A_{\eta }^{t-{\ell _s}} = w_1 = w_1 \cdot A^{t-\ell _s}$, and the term cancels out. Vector $w_2$ is over states of $R_*(s)$, with $\sum _{i \in R_*} w_2[i] \le (1-\text {Cond}_s^{\ell _s}(A))$, and we obtain an inductive term $\le (1-\text {Cond}_s^{\ell _s}(A)) E(t-\ell _s)$. Last, vector $w_3$ is over states not in *R*(*s*), and we have $w_3 \cdot A_{\eta }^{t-\ell _s} \cdot v_s = 0 = w_3 \cdot A^{t-\ell _s} \cdot v_s$, and the term cancels out.

We also obtain that $|v_j \cdot (A_{\eta }^{t}- A^{\ell _s} A_{\eta }^{t-\ell _s}) \cdot v_s|\le \ell _s \cdot \eta$. Thus, we have the inductive formula $E(t)\le (1-\text {Cond}_s^{\ell _s}(A)) E(t-\ell _s) + \ell _s \cdot \eta$. It yields for all $t \in \mathbb {N}$:

$$E(t) \le (\ell _s\cdot \eta )\sum _{i=1}^\infty (1-\text {Cond}_s^{\ell _s}(A))^i$$

$$E(t)\le \frac{\ell _s \cdot \eta }{Cond_s^{\ell _s}(A)}$$

$$\square $$

We can extend this result from reachability to formulas of the form $S_0 \mathbf {U} S_F$, where $S_0,S_F$ are subsets of states. This formula means that we reach the set of states $S_F$ through only states in $S_0$ on the way.

We define $R(S_0,S_F)$ to be the set of states which can reach $S_F$ using only states of $S_0$, and $R_*(S_0,S_F) = R(S_0,S_F) \setminus S_F$. For $\ell \in \mathbb {N}$, we let:

$$\begin{aligned} \text {Cond}^\ell _{S_0,S_F}(A)=\min _{i \in R_*(S_0,S_F)} \mathbb {P}^A_i(\mathbf {F}_{ \le \ell } \lnot R_*(S_0,S_F) \vee \lnot S_0). \end{aligned}$$

Now, one can remark that $\text {Cond}_{S_0,S_F}(A) \ge \text {Cond}_{S,S_F}(A)>0$. Let $\text {Cond}^\ell _{S_F}(A)=\text {Cond}^\ell _{S,S_F}(A)$. We have $\text {Cond}^\ell _{S_0,S_F}(A) \ge \text {Cond}^\ell _{S_F}(A)$. As before, we let $\ell _{S_F} \le m$ be the minimal $\ell$ such that $\text {Cond}^\ell _{S_F}(A)>0$, and obtain:

#### Theorem 5

Denoting $\varphi$ the property $S_0 \mathbf {U} S_F$, we have, given DTMC *A*:

$$\begin{aligned} |\gamma (A,\varphi )-\gamma (A_{\eta },\varphi )|< \frac{\ell _{S_F} \cdot \eta }{\text {Cond}^{\ell _{S_F}}_{S_F}(A)} \end{aligned}$$

We can actually improve this conditioning: we defined it as the probability to reach $S_F$ or $S \setminus R(S,S_F)$. At the price of a more technical proof, we can obtain a better bound by replacing $S_F$ by the set of states $R_1(S_F)$ that have probability 1 to reach $S_F$. We let $\overline{R_*}(S_F)=R(S,S_F) \setminus R_1(S_F)$ the set of states that can reach $S_F$ with $<1$ probability, and define the *refined conditioning* as follows:

$$\begin{aligned} \overline{\text {Cond}}_{S_F}^\ell (A)=\min _{i \in \overline{R_*}(S_F)} \mathbb {P}^A_i(\mathbf {F}_{\le \ell } \lnot \overline{R_*}(S_F)) \end{aligned}$$

### Optimality of the Conditioning

We show now that the bound we provide in Theorem 4 is close to optimal.

Consider again DTMCs $A,\hat{A}$ in Fig. 3 from example 1, and formula $\mathbf {F} \,s_2$ stating that $s_2$ is eventually reached. The probabilities to satisfy this formula in $A,\hat{A}$ are respectively $\mathbb {P}^{A}(\mathbf {F} \,s_2)= \frac{1}{2}$ and $\mathbb {P}^{\hat{A}}(\mathbf {F} \,s_2)= \frac{1}{2}-\frac{\eta }{4\tau }$. Assume that *A* is the real system and that $\hat{A}$ is the DTMC we learned from *A*.

As we do not know precisely the transition probabilities in *A*, we can only compute the conditioning on $\hat{A}$ and not on *A* (it suffices to swap *A* and $A_\eta$ in Theorem 4 and 5 to have the same formula using $\text {Cond}(A_\eta )=\text {Cond}(\hat{A})$). We have $R(s_2)=\{s_1,s_2\}$ and $R_*(s_2)=\overline{R_*}(s_2)=\{s_1\}$. The probability to stay in $R_*(s_2)$ after $\ell _{s_2}=1$ step is $(1-2\tau )$, and thus $\text {Cond}^1_{\{s_2\}}(\hat{A})= \overline{\text {Cond}}^1_{\{s_2\}}(\hat{A}) =1- (1-2\tau ) = 2 \tau$. Taking $A_{\eta }=\hat{A}$, Theorem 5 tells us that $|\mathbb {P}^{A}(\mathbf {F} \,s_2)-\mathbb {P}^{\hat{A}}(\mathbf {F} \,s_2)| \le \frac{\eta }{2 \tau }$. Notice that on that example, using $\ell _{s_2}=m=3$, we obtain $\text {Cond}^3_{\{s_2\}}(\hat{A})= 1- (1-2\tau )^3 \approx 6 \tau$, and we find a similar bound $\approx \frac{3 \eta }{6 \tau } = \frac{\eta }{2 \tau }$.

Compare our bound with the exact difference $|\mathbb {P}^{A}(\mathbf {F} \,s_2)-\mathbb {P}^{\hat{A}}(\mathbf {F} \,s_2)| = \frac{1}{2} - (\frac{1}{2}-\frac{\eta }{4\tau })= \frac{\eta }{4\tau }$. Our upper bound only has an overhead factor of 2, even while the conditioning is particularly bad (small) in this example.

### PAC Bounds for $\sum _j |\hat{A}_W(i,j) - A(i,j)| \le \eta$

We use Theorem 1 in order to obtain PAC bounds. We use it to estimate individual transition probabilities, rather than the probability of a property.

Let *W* be a set of traces drawn with respect to *A* such that every $\omega \in W$ is of the form $\omega = \rho \cdot s \cdot \rho ' \cdot s$. Recall for each state *i*, *j* of *S*, $n^W_i$ is the number of transitions originating from *i* in *W* and $n^W_{ij}$ is the number of transitions $ss'$ in *W*. Let $\delta ' = \frac{\delta }{m_\text {stoch}}$, where $m_{\text {stoch}}$ is the number of *stochastic* states, i.e., with at least two outgoing transitions.

We want to sample traces until the empirical transition probabilities $\frac{n^W_{ij}}{n^W_i}$ are relatively close to the exact transition probabilities $a_{ij}$, for all $i,j\in S$. For that, we need to determine a stopping criteria over the number of state occurrences $(n_i)_{1\le i\le m}$ such that:

$$\begin{aligned} \mathbb {P}\left( \exists i \in S,\, \sum _j \left| a_{ij} - \frac{n^W_{ij}}{n^W_i}\right| > \varepsilon \right) \le \delta \end{aligned}$$

First, note that for any observed state $i\in S$, if $a_{ij}=0$ (or $a_{ij}=1$), then with probability 1, $\frac{n^W_{ij}}{n^W_i}=0$ (respectively $\frac{n^W_{ij}}{n^W_i}=1$). Thus, for all $\varepsilon >0$, $|a_{ij}-\frac{n^W_{ij}}{n^W_i}|<\varepsilon$ with probability 1. Second, for two distinct states *i* and $i'$, the transition probabilities $\frac{n^W_{ij}}{n^W_i}$ and $\frac{n^W_{i'j'}}{n^W_{i'}}$ are independent for all $j,j'$.

Let $i\in S$ be a stochastic state. If we observe $n^W_i$ transitions from *i* such that $n^W_i\ge \frac{2}{\varepsilon ^2}\log \left( \frac{2}{\delta '}\right) \left[ \frac{1}{4}-\left( \max _{j}|\frac{1}{2}-\frac{n^W_{ij}}{n^W_i}|-\frac{2}{3}\varepsilon \right) ^2\right]$, then, according to Theorem 1, $\mathbb {P}\left( \bigvee _{j=1}^m\, |a_{ij} - \frac{n^W_{ij}}{n^W_i}| > \varepsilon \right) \le \delta '$. In particular, $\mathbb {P}\left( \max _{j\in S}|a_{ij} - \frac{n^W_{ij}}{n^W_i}| > \varepsilon \right) \le \delta '$. Moreover, we have:

$$\begin{aligned} \mathbb {P}\left( \bigvee _{j=1}^m\, \max _{j\in S}|a_{ij} - \frac{n^W_{ij}}{n^W_i}|> \varepsilon \right)&\le \sum _{j=1}^m \mathbb {P}\left( \max _{j\in S}|a_{ij} - \frac{n^W_{ij}}{n^W_i}| > \varepsilon \right) \\&\le m_\text {stoch} \delta '\\&\le \delta \end{aligned}$$

In other words, the probability that “there exists a state $i\in S$ such that the deviation between the exact and empirical outgoing transitions from *i* exceeds $\varepsilon$” is bounded by $\delta$ as soon as for each state $i\in S$, $n^W_i$ satisfies the stopping rule of the algorithm of Chen using $\varepsilon$ and the corresponding $\delta '$. This gives the hypothesis $\sum _j |A_{\eta }(i,j) - A(i,j)| \le \epsilon$ for all states *i* of Sect. 6.2.

### A Matrix $\hat{A}_W$ Accurate for all CTL properties

We now use Laplace smoothing in order to ensure the other hypothesis $A_{\eta }(i,j) \ne 0$ iff $A(i,j) \ne 0$ for all states *i*, *j*. For all $i \in S$, we define the Laplace offset depending on the state *i* as $\alpha _i=\frac{(n^W_i)^2 \varepsilon }{10 \cdot k_i^2 \max _j n^W_{ij}}$, where $k_i$ is the number of transitions from state *i*. This ensures that the error from Laplace smoothing is at most one tenth of the statistical error. Let $\alpha =(\alpha _i)_{1 \le i \le m}$. From the sample set *W*, we output the matrix $\hat{A}^\alpha _W=(\hat{a}_{ij})_{1 \le i,j \le m}$ with Laplace smoothing $\alpha _i$ for state *i*, i.e.:

$$\hat{a}_{ij}= \frac{n^W_{ij}+\alpha _i}{n^W_i+k_i \alpha _i} \text { if } a_{ij} \ne 0 \quad \text { and } \quad \hat{a}_{ij}=0 \text { otherwise}$$

It is easy to check that we have for all $i,j \in S$: $\left| \hat{a}_{ij} - \frac{n^W_{ij}}{n^W_i}\right| \le \frac{\varepsilon }{10 \cdot k_i}$

That is, for all states *i*, $\sum _j \left| \hat{a}_{ij} - \frac{n^W_{ij}}{n^W_i}\right| \le \frac{\varepsilon }{10}$. Using the triangular inequality:

$$\begin{aligned} \mathbb {P}\left( \exists i\in S, \sum _j \left| a_{ij} - \hat{a}_{ij}\right| > \frac{11}{10}\varepsilon \right) \le \delta \end{aligned}$$

For all $i\in S$, let $H^*(n^W_i,\epsilon ,\delta ')=\max _{j\in S}H(n^W_i,n^W_{ij},\epsilon ,\delta ')$ be the maximal Chen bound over all the transitions from state *i*. Let $B(\hat{A}^{\alpha }_W)=\max _{S_F} \frac{\ell _{S_F}}{\overline{\text {Cond}}_{S_F}^{\ell _{S_F}}(\hat{A}^{\alpha }_W)}$. Since in Theorem 5, the original model and the learned one have symmetric roles, by applying this theorem on $\hat{A}^{\alpha }_W$, we obtain that:

#### Theorem 6

Given a set *W* of traces, for $0<\epsilon <1$ and $0<\delta <1$, if for all $i\in S$, $n^W_i\ge \left( \frac{11}{10}B(\hat{A}^{\alpha }_W)\right) ^2 H^*(n^W_i,\epsilon ,\delta ')$, we have for any CTL property $\varphi$:

9 $$\begin{aligned} \mathbb {P}(|\gamma (A,\varphi ) - \gamma (\hat{A}^{\alpha }_W,\varphi )|)> \varepsilon )\le \delta \end{aligned}$$

#### Proof

First, $\hat{a}_{ij} \ne 0$ iff $a_{ij} \ne 0$, by definition of $\hat{A}^{\alpha }_W$. Second, $\mathbb {P}( \exists i, \sum _j |a_{ij} - \hat{a}_{ij}| > \frac{11}{10}\varepsilon )\le \delta$. We can thus apply Theorem 5 on $\hat{A}^\alpha _W, A$ and obtain (9) for $\varphi$ any formula of the form $S_1 \mathbf {U} S_2$. It remains to show that for any formula $\varphi \in \varPsi$, we can define $S_1,S_2 \subseteq S$ such that $\varphi$ can be expressed as $S_1 \mathbf {U} S_2$.

Consider the different cases: If $\varphi$ is of the form $\varphi =\varphi _1 \mathbf {U} \varphi _2$ (it subsumes the case $\varphi =\mathbf {F} \varphi _1 = \top \mathbf {U} \varphi _1$) with $\varphi _1,\varphi _2$ CTL formulas, we define $S_1,S_2$ as the sets of states satisfying $\varphi _1$ and $\varphi _2$, and we have the equivalence (see [2] for more details). If $\varphi =X \varphi _2$, define $S_1=\emptyset$ and $S_2$ as the set of states satisfying $\varphi _2$.

The last case is $\varphi =\mathbf {G} \varphi _1$, with $\varphi _1$ a CTL formula. Again, we define $S_1$ the set of states satisfying $\varphi _1$, and $S_2$ the set of states satisfying the CTL formula $\mathbf {AG} \varphi _1$. The probability of the set of paths satisfying $\varphi =\mathbf {G} \varphi _1$ is exactly the same as the probability of the set of paths satisfying $S_1 \mathbf {U} S_2$.$\square$


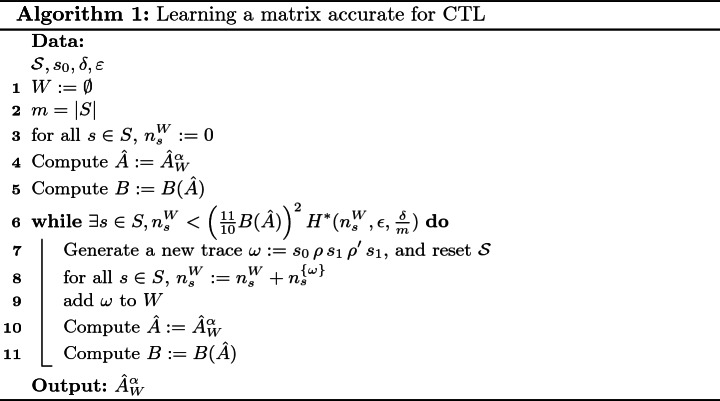


### Algorithm

We give more details about the learning process of a Markov Chain, accurate for every CTL formula. For completeness, we also provide in the extended version a similar algorithm for a time-to-failure property.

A path $\omega$ is observed from $s_0$ till a state is observed twice. Then $\omega$ is added to *W* and the reset operation is performed. We use Laplace smoothing to compute the corresponding matrix $\hat{A}^\alpha _W$. The error bound is computed on *W*, and a new path $\omega '$ is then being generated if the error bound is not as small as desired.

This algorithm is guaranteed to terminate since, as traces are generated, with probability 1, $n^W_s$ tends towards $\infty$, $\hat{A}^\alpha _W$ tends towards *A*, and $B(\hat{A}^\alpha _W)$ tends towards *B*(*A*).

## Evaluation and Discussion

In this section, we first evaluate Algorithm 1 on 5 systems which are crafted to evaluate the algorithm under different conditions (e.g., rare states). The objective of the evaluation is to provide some idea on how many samples would be sufficient for learning accurate DTMC estimations, and compare learning for all properties of CTL and learning for one time-to-failure property.

Then, we evaluate our algorithm on very large PRISM systems (millions or billions of states). Because of the number of states, we cannot learn a DTMC accurate for all properties of CTL there: it would ask to visit every single state a number of times. However, we can learn a DTMC for one specific (unbounded) property. We compare with an hypothesis testing algorithm from [31] which can handle the same unbounded property through a reachability analysis using the topology of the system.

**Table 1.** Average number of observed events *N* (and relative standard deviation in parenthesis) given $\varepsilon =0.1$ and $\delta =0.05$ for a time-to-failure property and for the full CTL logic using the refined conditioning $\overline{\text {Cond}}$.

	System 1	System 2	System 3	System 4	System 5
$\#$ states	3	3	30	64	200
$\#$ transitions	4	7	900	204	40, 000
$\#$ events for time-to-failure	$191 \, (16\%)$	$991 \, (10\%)$	$2{,}753 \, (7.4\%)$	$1{,}386 \, (17.9\%)$	$18{,}335 \, (7.2\%)$
$\#$ events for full CTL	$1{,}463 \, (12.9\%)$	$4{,}159 \, (11.7\%)$	$8{,}404 \, (3.8\%)$	1, 872, 863	$79{,}823 \, (1.7\%)$

### Evaluation on Crafted Models

We first describe the 5 systems: Systems 1 and 2 are three-state models described in Fig. 1 and Fig. 2. Systems 3 (resp. 5) is a 30-state (resp. 200-states) clique in which every individual transition probability is 1/30 (resp. 1/200). System 4 is a 64-state system modeling failure and repair of 3 types of components (3 components each, 9 components in total), see the extended version for a full description of the system, including a PRISM [20] model for the readers interested to investigate this system in details.

We tested time-to-failure properties by choosing as failure states $s_3$ for Systems 1, 2, 3, 5, and the state where all 9 components fail for System 4. We also tested Algorithm 1 (for full CTL logic) using the refined conditioning $\overline{\text {Cond}}$. We performed our algorithms 100 times for each model, except for full CTL on System 4, for which we only tested once since it is very time-consuming. We report our results in Table 1 for $\varepsilon =0.1$ and $\delta =0.05$. In particular, we output for each model its number of states and transitions. For each (set of) property, we provide the average number of observations (i.e. the number of samples times their average length) and the relative standard deviation (in parenthesis, that is the standard deviation divided by the average number of observed events).

The results show that we can learn a DTMC with more than 40000 stochastic transitions, such that the DTMC is accurate for all CTL formulas. Notice that for some particular systems such as System 4, it can take a lot of events to be observed before Algorithm 1 terminates. The reason is the presence of rare states, such as the state where all 9 components fail, which are observed with an extremely small probability. In order to evaluate the probabilities of CTL properties of the form: “if all 9 components fail, then CTL property $\varphi$ is satisfied”, this state needs to be explored many times, explaining the high number of events observed before the algorithm terminates. On the other hand, for properties that do not involve the 9 components failing as prior, such as time-to-failure, one does not need to observe this state even once to conclude that it has an extremely small probability to happen. This suggests that efficient algorithms could be developed for subsets of CTL formulas, e.g., in defining a subset of important events to consider. We believe that Theorem 4 and 5 could be extended to handle such cases. Over different runs, the results stay similar (notice the rather small relative standard deviation).

Comparing results for time-to-failure (or equivalently SMC) and for the full CTL logic is interesting. Excluding System 4 which involves rare states, the number of events that needs to be observed for the full CTL logic is 4.3 to 7 times more. Surprisingly, the highest difference is obtained on the smallest System 1. It is because every run of System 1 generated for time-to-failure is short ($s_1 s_2 s_1$ and $s_1 s_2 s_3$). However, in Systems 2,3 and 5, samples for time-to-failure can be much longer, and the performances for time-to-failure (or equivalently SMC) is not so much better than for learning a DTMC accurate for all CTL properties.

For the systems we tested, the unoptimized $\text {Cond}$ was particularly large (more than 20) because for many states *s*, there was probability 0 to leave *R*(*s*), and hence $\ell (s)$ was quite large. These are the cases where $\overline{\text {Cond}}$ is much more efficient, as then we can choose $\ell _s=1$ as the probability to reach *s* from states in *R*(*s*) is 1 ($R_1(s)=R(s)$ and $\overline{R_*(s)}=\emptyset$). We used $\overline{\text {Cond}}$ in our algorithm.

Finally, we evaluate experimental confidence by comparing the time-to-failure probabilities in the learned DTMC and the original system. We repeat our algorithms 1000 times on System 1 and 2 (with $\varepsilon =0.1$ and $\delta =0.05$). These probabilities differ by less than $\varepsilon$, respectively 999 and 995 times out of 1000. Specification (2) is thus largely fulfilled (the specification should be ensured 950 out of 1000 times), that empirically endorses our approach. Hence, while our PAC bound over-approximates the confidence in the learned system (which is unavoidable), it is not that far from experimental values.

### Evaluation on Large Models

We also evaluated our algorithm on large PRISM models, ranging from hundreds of thousands to billions of states. With these numbers of states, we cannot use the more ambitious learning over all the properties of CTL, which would need to visit every states a number of times. However, we can use our algorithm for learning a DTMC which is accurate given a particular (unbounded) property: it will visit only a fraction of the states, which is enough to give a model accurate for that property, with a well-learned kernel of states and some other states representatives for the remaining of the runs. We consider three test-cases from PRISM, satisfying the property that the sample stops with a conclusion (yes or no) with probability 1. Namely, *herman, leader* and *egl*.

**Table 2.** Results for $\varepsilon =0.01$ and $\delta =0.001$ of our algorithm compared with sampling with reachability analysis [31], as reported in [14], page 20. Numbers of samples needed by our method are given by the Massart bound (resp. by the Okamoto-Chernoff bound in parenthesis). TO and MO means time out ($>15$ minutes on an Opteron 6134) and memory out ($>5$GB) respectively.

Model name	Size	Our learning method		Sampling with reachability analysis [31]	
		Samples	Path length	Samples	Path length
herman(17)	129M	506 (38K)	27	219	30
herman(19)	1162M	506 (38K)	40	219	38
herman(21)	10G	506 (38K)	43	219	48
leader(6, 6)	280K	506 (38K)	7.4	219	7
leader(6, 8)	${>}280$K	506 (38K)	7.4	(MO)	(MO)
leader(6, 11)	${>}280$K	506 (38K)	7.3	(MO)	(MO)
egl(15, 10)	616G	38K (38K)	470	1100	201
egl(20, 15)	1279T	38K (38K)	930	999	347
egl(20, 20)	1719T	38K (38K)	1200	(TO)	(TO)

Our prototype tool used in the previous subsection is implemented in Scilab: it cannot simulate very large systems of PRISM. Instead, we use PRISM to generate the samples needed for the learning. Hence, we report the usual Okamoto-Chernoff bound on the number of samples, which is what is implemented in PRISM. We also compare with the Massart bound used by the Chen algorithm (see Sect. 2.2), which is implemented in our tool and is more efficient as it takes into account the probability of the property.

For each model, we report its parameters, its *size*, i.e. its number of states, the number of *samples* needed using the Massart bound (the conservative Okamoto-Chernoff bound is in parenthesis), and the average *path length*. For comparison, we consider an hypothesis testing algorithm from [31] which can also handle unbounded properties. It uses the knowledge of the topology to do reachability analysis to stop the sampling if the property cannot be reached anymore. Hypothesis testing is used to decide with high confidence whether a probability exceeds a threshold or not. This requires less samples than SMC algorithms which estimate probabilities, but it is also less precise. We chose to compare with this algorithm because as in our work, it does not require knowledge on the probabilities, such as a lower bound on the transition probabilities needed by e.g. [14]. We do not report runtime as they cannot be compared (different platforms, different nature of result, etc.).

There are several conclusions we can draw from the experimental results (shown in Table 2). First, the number of samples from our algorithm (Chen algorithm implementing the Massart bound) are larger than in the algorithm from [31]. This is because they do hypothesis testing, which requires less samples than even estimating the probability of a property, while we learn a DTMC accurate for this property. For *herman* and *leader*, the difference is small (2.5x), because it is a case where the Massart bound is very efficient (80 times better than Okamoto-Chernoff implemented in PRISM). The *egl* system is the worst-case for the Massart bound (the probability of the property is $\frac{1}{2}$), and it coincides with Okamoto-Chernoff. The difference with [31] is 40x in that case. Also, as shown in *egl*, paths in our algorithm can be a bit larger than in the algorithm from [31], where they can be stopped early by the reachability analysis. However, the differences are never larger than 3x. On the other hand, we learn a model representative of the original system for a given property, while [31] only provide a yes/no answer to hypothesis testing (performing SMC evaluating the probability of a property with the Massart bound would give exactly the same number of samples as we report for our learning algorithm). Last, the reachability analysis from [31] does time out or memory out on some complex systems, which is not the case with our algorithm.

## Conclusion

In this paper, we provided theoretical grounds for obtaining global PAC bounds when learning a DTMC: we bound the error made between the behaviors of the model and of the system, formalized using temporal logics. While it is not possible to obtain a learning framework for LTL properties, we provide it for the whole CTL logic. For subsets of CTL, e.g. for a fixed timed-to-failure property, we obtain better bounds, as efficient as Statistical MC. Overall, this work should help in the recent trends of establishing trusted machine learning [16].

Our techniques are useful for designers of systems for which probabilities are governed by uncertain forces (e.g. error rates): in this case, it is not easy to have a lower bound on the minimal transition probability, but we can assume that the set of transitions is known. Technically, our techniques provides rationale to set the constant in Laplace smoothing, otherwise left to an expert to set.

Some cases remain problematic, such as systems where states are visited very rarely. Nevertheless, we foresee potential solutions involving rare event simulation [21]. This goes beyond the scope of this work and it is left to future work.

## Footnotes

1 We recall the Okamoto-Chernoff bound in the extended version (as well as the Massart bound), but we do not use it in this work.
